# Alteration of the −35 and −10 sequences and deletion the upstream sequence of the −35 region of the promoter A1 of the phage T7 in dsDNA confirm the contribution of non-specific interactions with *E. coli* RNA polymerase to the transcription initiation process

**DOI:** 10.3389/fmolb.2023.1335409

**Published:** 2024-01-08

**Authors:** Katarzyna Turecka, Małgorzata Firczuk, Władysław Werel

**Affiliations:** ^1^ Department of Pharmaceutical Microbiology, Faculty of Pharmacy, Medical University of Gdańsk, Gdańsk, Poland; ^2^ Laboratory of Immunology, Mossakowski Medical Research Institute, Polish Academy of Sciences, Warsaw, Poland

**Keywords:** −35 region, −10 region, upstream sequence, non-specific interactions, promoter A1 of the phage T7, transcription initiation

## Abstract

Transcription initiation is a multi-step process, in which the RNA polymerase holoenzyme binds to the specific promoter sequences to form a closed complex, which, through intermediate stages, isomerizes into an open complex capable of initiating the productive phase of transcription. The aim of this work was to determine the contribution of the −10 and −35 regions of the promoter, as well as the role of non-specific interactions, in the binding of RNA polymerase and the formation of an active initiation complex capable of transcription. Therefore, fragments of promoter DNA, derived from the strong promoter A1 of the phage T7, containing completely and partially altered elements −35 and −10, and devoid of an upstream region, were constructed using genetic engineering methods. Functional analyses of modified promoter fragments were carried out, checking their ability to form binary complexes with *Escherichia coli* RNA polymerase (RNAP) and the efficiency of converting binary complexes into triple complexes characteristic of the productive phase of transcription. The obtained results suggest that, in relation to the A1 promoter of the T7 phage, the most important role of the −35 region is carrying the open complex through the next phases of transcription initiation. The weakening of specific impacts within the region −35 is a reason for the defect associated with the transformation of the open complex, formed by a DNA fragment containing the completely altered −35 region, into elongation and the impairment of RNA synthesis. This leads to breaking contacts with the RNA polymerase holoenzyme, and destabilization and disintegration of the complex in the initial phase of productive transcription. This confirms the hypothesis of the so-called stressed intermediate state associated with the stage of transition from the open complex to the elongation complex. The experiments carried out in this work confirm also that the process of promoter localization and recognition, as well as the formation of binary complexes, is sequential in nature, and that the region located upstream of the −35 hexamer, and the hexamer itself, plays here an additive role.

## 1 Introduction

Promoters are DNA segments containing signals responsible for proper binding and subsequent activation of the RNA polymerase holoenzyme (RNAP, composed of 6 subunits, 2 α, β, β’, ω, and σ), an enzyme that catalyzes the transcription process (Borukhov and Nudler, 2008; Sutherland and Murakami, 2018) into a form capable of initiating RNA synthesis (von Hippel. et al., 1984).

Analyses of promoter sequence interactions with RNA polymerase have shown that the enzyme recognized promoter sequences through sequence-specific interactions of the sigma factor with highly conserved −35 and −10 regions (for σ^70^-dependent promoters) (Siebenlist and Gilbert, 1980; Campbell et al., 2002; Feklistov and Darst, 2011; Campagne et al., 2014; Feklistov et al., 2014). Other promoter elements are also important in the transcription initiation process, namely, the length and nucleotide content of the spacer region (located between −10 and −35 elements) (Liu et al., 2004; Klein et al., 2021), an UP element upstream of the −35 (Estrem et al., 1998), and the sequences surrounding these elements (Carr et al., 2017), including G/C rich-“discriminator” of 6–8 bp, located between the −10 element and the transcription start site (TSS) (Shimada et al., 2014; Forquet et al., 2021). The importance of −35 and −10 hexamers through mutations concentrated in these areas, which significantly affect the activity of the promoter, was largely highlighted (Stefano and Gralla, 1982; Youderian et al., 1982; Hawley and McClure, 1983; Vo et al., 2003). Promoter DNA sequences upstream of the −35 region, the −35 region, and a portion of the −10 elementaffect initial binding, subsequent isomerizations, and rates of open complex (RPo) formation. Whereas sequences in the −10 element and further downstream have larger effects on RPo stability (Ruff et al., 2015). The −35 element has the consensus sequence 5′-TTGACA-3' (Hawley and McClure, 1983) with −35T, −34T, and −33G being the most highly conserved (Hawley and McClure, 1983). It was shown that the −35 element interacts with the σ4 domain primarily through the insertion of a helix-turn-helix (HTH) into the DNA major groove; protein-DNA interaction extends on the non-template strand from −35 to −38 and on the template strand from −31 to −33 (Campbell et al., 2002; Murakami et al., 2002; Zuo and Steitz, 2015). Moreover, an α-subunit C-terminal domain (α-CTD) interacts with the DNA minor grove from −43 to −38 and binds just upstream of the σ^70^4 (Ross et al., 1993; Benoff et al., 2002; Ross and Gourse, 2005). The extensive contacts between promoter fragment and σ4 cause significant DNA bending by about 30° (Zuo and Steitz, 2015).

It was demonstrated that the substitutions at position −32 and −34 of the −35 region had the greatest influence on promoter strength, while substitutions at position −31 had no significant effect on this parameter. Substitution of the −32 nucleotide drastically reduced the affinity of the RNA polymerase for the promoter; the base at this position is directly involved in binding to RNA polymerase (region 4.2 of the sigma 70 subunit). Substitutions at the −33 position had a different effect depending on which base was introduced. Insertion of a guanine generated the TTG trimer, which is conserved in many promoters and is characteristic of the strongest promoters. An increase in affinity occurs also when guanine is introduced at position −34, while incorporation of cytosine or adenine generates the weakest promoters (Vo et al., 2003). Regardless of the important role assigned to the −35 hexamer, it was presented that the sequence of the −35 region can be successfully replaced by contacts of RNA polymerase with appropriate protein activators, or by the presence of an additional conserved sequence near the −10 region, the so-called extended −10 promoters (Bown et al., 1999).

Siebenlist was the first to show that the formation of the open complex (in the A3 promoter of phage T7) is combined with strand separation in the region spanning positions −8 to +3, thus overlapping the −10 region (Siebenlist and Gilbert, 1980). Using *in vitro* mutagenesis, it was demonstrated that the −10 hexamer is very important at the stage of the recognition of double-stranded DNA, closed complex formation, stabilizing the initial interactions of the double-stranded DNA form with RNA polymerase, and then, as single-stranded DNA, it directs the process of isomerization of this enzyme to its active, functional heparin-resistant form. Any substitutions in the −10 region when the DNA is “open” are of little importance, indicating that when the DNA is in a single-stranded form, the sequence is of little or no importance (Fenton and Gralla, 2001; Darst et al., 2002; Saecker et al., 2002). However, Saecker et al. (2021), using cryo-electron microscopy, showed that small changes in the sequence or length of the transcription bubble just upstream of the start site (+1) cause drastic changes in the DNA–RNAP interactions and base stacking (Saecker et al., 2021). Moreover, Chen et al. (2020) found that unpairing and unstacking the base pair at −12, which then repairs and restacks with −13 in the subsequent intermediate states, facilitates DNA entry into the active site channel of RNAP (Chen et al., 2020). In turn, Heyduk and Heyduk (2014) indicated a dominant role of consensus bases at positions −11 and −7, emphasizing a preference for A in −11 and T in −7 position, exhibiting the fastest melting kinetics and efficient promoter melting, respectively (Heyduk and Heyduk, 2014). The bases of the −11A (nt) and –7A (nt) residues are clearly flipped out, and the σ2 domain interacts with the −10 (nt-strain) (Feklistov and Darst, 2011; Zhang et al., 2012; Zuo and Steitz, 2015). The −10 (t-strand) wraps around the σ3 domain and passes through a cleft between σ2 and σ3 (Zuo and Steitz, 2015).

The results conducted by Bae et al. (2015) clearly show that Taq σA W256 (Eco σ70 W433) plays a role in the promoter opening pathway, which, rotating into dsDNA, serves as a steric mimic of the flipped out −11A (nt) base by a stacking mechanism (Bae et al., 2015). They also showed that Taq σA Y217 plays an important role in the promoter opening process, possibly by stacking with −11T (t), when the −11A (nt) base flips out. Kinetic studies indicate that W256 and Y217 act to increase the rate of open promoter step (Bae et al., 2015). −11 AT is probably the first base pair disrupted in the promoter opening pathway (Chen and Helmann, 1997; Lim et al., 2001; Heyduk et al., 2006; Feklistov and Darst, 2011). Substitutions at the highly conserved base positions −12T (in 80% of known promoter sequences) and –7T in single-stranded DNA had no effect on recognition by RNA polymerase (Qiu and Helmann, 1999). The T-A pair at the −12 position is characteristic for promoters with high transcriptional activity, from which the transcription process proceeds faster. However, despite the effective formation of an open complex with this type of promoter, fewer transcripts were formed in the *in vitro* transcription process under conditions of enzyme excess than in the case of the presence of the −12 position of the G-C pair (Xu et al., 2001). The G-C pair is only found in 4%–10% of naturally occurring, known promoters; however, many strong promoters contain this pair at this position, such as the λPr or T7A1 promoter. In addition, approximately 75% of the promoters containing a C-G base pair at the −10 position were also found to contain a G-C base pair at the −12 position (Xu et al., 2001). The most highly conserved base in the −10 region is the T at the −7 position (found in approximately 90% of known promoters).

Promoter sequences have developed in the process of evolution, but it is not possible for promoters to contain the optimal sequence for all stages of the complex process of transcription initiation. The presence of certain bases may, on the one hand, reduce the probability of forming a heparin-resistant binary complex, but on the other hand, it may facilitate isomerization of this complex into a form capable of RNA synthesis. Thus, the base sequence of the −10 region of the promoter has different effects on the various steps leading to the formation of an active transcription complex (Xu et al., 2001). Moreover, the base sequence that is important for the function of the promoter as a whole must be separated from the sequences that are important for single-stranded DNA binding.

Therefore, the aim of this work was to assess the contribution of the −10 and −35 regions of the promoter, as well as the role of non-specific interactions, in the binding of RNA polymerase and the formation of an active initiation complex capable of transcription. For this purpose, using the A1 promoter of phage T7, fragments of promoter DNA containing partially and completely changed regions −10 and −35 were constructed, analyzing the effectiveness of RNA polymerase binding by modified promoter sequences, the formation of open complexes, and the ability to enter the productive phase of transcription using the reaction of *in vitro* transcription under substrate limiting conditions.

## 2 Materials and methods

### 2.1 Bacterial strains, plasmids, media, and growth conditions

The DH5α [F-, Φ 80 lacZΔM15, recA1, gyrA96, thi-1, hsdR17, (rk-, mk-), supE44, re1A1deoR, Δ(lacZYA-argF), U169 (Z. Burton, Michigan State, University East Lansing)] strain of *Escherichia coli* was stored as glycerol stock at −70°C, and for research purposes the culture was kept at 37°C for 24 h in LB broth (Biomaxima, Poland). In this work, plasmid pDS1 (2,833 bp, [AmpR; ori-pBR322; ori-ColE1] (NCBI—490681; courtesy of H. Heumann) was also applied.

### 2.2 Polymerase chain reaction (PCR) of the wild-type and modified fragments of the phage T7 A1 promoter

The following PCR primers at a concentration of 10 µM to obtain the A1 promoter of phage T7 and one of the modified versions were used (Thermo Hybaid GmbH, Heidelberg, Germany): PLA1: 5′-GGA TCC TCG AGA TCC CGA AAA TTT ATC AAA-3’; PPA1: 5′-GGA TCC TCG AGC TCC AGA TCC CGG ACC C-3’; oligoLA1: 5′-GGA TCC CGA AAA TTT ATC AAA AAG AGT AGA ATT CTA AAG TCT AAC CTA TAG GAT ACTTAC-3’; TBL: 5′-GCG GAG ATC TGC CAT CGA GAG GGA C-3’; TBR1: 5′-GCG GAG ATC TCT GCG TAT AGG TTA GAG TTT A-3’; TBR2: 5′-GCG GAG ATC TCA TCC TAT AGG TTA GAC TT A-3’; kfA1: 5′-GTA TTG ACT TAA AGT CTA ACC TAT AG-3’; kfO-35: 5′-GTA GAA TTC TAA AGT CTA ACC TAT AG-3’. PCR reaction products were evaluated using 1.5% agarose gel electrophoresis in the presence of 1x concentrated TAE buffer (Tris, acetic acid, EDTA, pH 8.3) (Sigma Technology, Poland).

### 2.3 Preparation of T7 phage A1 promoter fragments altered in the −35 and −10 regions

To obtain fragments of the T7 phage A1 promoter altered in the −35 and −10 regions, a PCR reaction was performed using the appropriate primers (Section 2.2). This enabled the introduction of an additional restriction site, *Bgl*II to fragments containing the altered −10 region and *Eco*RI to the fragment containing the altered −35 region.

### 2.4 Labelling of primers with [γ-^32^P] ATP

Samples for the labelling reaction were prepared as follows: 40 µL of the 10 µM primer was mixed with 10 µL of T4 phage kinase buffer (10 × concentrated). Then 5 µL of [γ-^32^P] ATP (specific activity 7,000 Ci/mol), 6 µL of polynucleotide kinase (10 u/µL) were added to the mixture and made up to 100 µL with sterile deionized water. All ingredients were thoroughly mixed and incubated for 30–45 min at 37°C. The enzyme was then inactivated for 10 min at 68°C and the primers were purified with the AIQuick Nucleotide Removal Kit according to the manufacturer’s instructions (QIAquick, 1997). In the case of ApUpC, no purification was performed after enzyme inactivation.

### 2.5 Study of interactions between DNA and RNA polymerase - the reaction of binary complexes formation

Binary complexes were prepared according to the following scheme (Table 1; Table 2). The RNA polymerase holoenzyme used in the study, was originated from Thermo Fisher Scientific, United States.

**TABLE 1:** Preparation of binary complexes formed by *E. coli* RNA polymerase with various fragments of the A1 promoter of the phage T7.

	A1_WT_ (89 ng/µL)	A1_−10_ (40 ng/µL)	A1_1/2−10_ (38 ng/µL)	A1_−35_ (87 ng/µL)
DNA labeled with ^32^P	1.0	2.2	2.3	1.0
Polymerase RNA holoenzyme (4 μg/mL)	5.5	5.5	5.5	5.5
Buffer for complexes 10 × concentrated	1.0	1.0	1.0	1.0
Water	2.5	1.3	1.2	2.5

**TABLE 2:** Preparation of binary complexes formed by *E. coli* RNA polymerase and various fragments of the A1 promoter of the phage T7.

	A1_−35/−10_ (35 ng/µL)	A1_−35/1/2−10_ (36 ng/µL)	sA1_WT_ (35 ng/µL)	sA1_−35_ (96 ng/µL)
DNA labeled with ^32^P	2.5	2.5	2.5	1.0
Polymerase RNA holoenzyme (4 μg/mL)	5.5	5.5	5.5	5.5
Buffer for complexes 10 × concentrated	1.0	1.0	1.0	1.0
Water	1.0	1.0	1.0	2.5

The prepared reaction mixtures were incubated for 20 min at 37°C and then dialyzed against TE buffer (1M Tris-HCl pH 7.9, 0.5M EDTA pH 8.0, redistilled water to 1,000 µL) at room temperature for 1 h (Milipore VS dialysis membranes with a pore diameter of 0.025 µm were used). After dialysis, 1 µL of heparin was added to the samples to give a final concentration of 1 mg/mL, and 1 µL of 6x concentrated electrophoresis dyes solution (0.09% bromophenol blue, 0.09% xylene cyanide, 60% glycerol and 60 mM EDTA). The whole probes were then loaded on a 3.5% native polyacrylamide gel and electrophoresis in TBE buffer (Tris, boric acid, 0.5M EDTA pH 8.0, deionized water up to 1,000 mL and HCl to pH 8.3) at 70V for about 2.5–3 h was performed.

### 2.6 *In vitro* transcription

5 µL of the MIX mixture (ApUpC, ATP, GTP, MgCl_2_) was added to the binary complexes and incubated for 20 min at 37°C. After incubation, 1 µL of heparin, 2 µL of a 6x concentrated electrophoresis dye solution were added and gel loaded. Electrophoresis was carried out as in Section 2.5. For the transcription reaction that used labelled ApUpC, unlabelled DNA was used.

### 2.7 Cleavage of binary complexes with restriction enzymes

To 10 µL of samples containing binary complexes, 1 µL of heparin at a concentration of 10 mg/mL was added, and then digestion with the appropriate restriction enzyme was performed: complexes of fragments A1_−10_ and A1_1/2−10_ with RNA polymerase holoenzyme were digested with *Bgl*II, fragment A1_−35_ with RNA polymerase holoenzyme was digested with the *Eco*RI enzyme, while the A1_−35/−10_ and A1_−35/1/2–−10_ fragments complexes were digested with the *Eco*RI and *Bgl*II enzymes 37°C for 3 h. The digested complexes were then applied to previously washed 2 mL TE buffer nitrocellulose membrane filters with a pore diameter of 0.45 µm, filtered and washed again with 2 mL of the same buffer. The complexes immobilized onto Millipore filters were placed in 200 µL of buffer containing 1% SDS and 0.3 M sodium acetate. DNA was eluted from the filters for 3 h (or overnight) at 37°C. The eluted DNA was then precipitated with 96% ethyl alcohol and resuspended in 10 µL deionized sterile water. Finally, DNA was analyzed on a 6% polyacrylamide gel under non-denaturing conditions (1x concentrated TBE, 120V, 60–70 min).

### 2.8 Footprint using potassium permanganate (KMnO_4_)

10 µL of the binary complexes solutions were dialyzed against TE buffer for 1 hour at room temperature. After dialysis, 1 µL of heparin with a starting concentration of 20 mg/mL was atteched to the samples (about 20 µL) and after 20 s 1.5 µL of a 15 mM potassium permanganate solution was added. The reaction was performed at 37°C for 1 min, then 1.5 µL of β-mercaptoethanol was added to complete the reaction. Samples were made up to 100 µL with water and 200 µL of phenol and next chloroform were added. Mixed thoroughly and centrifuged for 5 min at 15,850 × g. The top layer (about 100 µL) was collected and 20 µL of 13M ammonium acetate was added, mixed thoroughly, then 400 µL of cold (0°C) 96% ethyl alcohol was added. Next, probes were incubated for 30 min at - 20 C, centrifuged 10 min at 15,850 × g and then washed twice with 70% ethyl alcohol. The resulting precipitates were dried under vacuum for 10 min, and then dissolved in 63 µL of sterile, deionized water. Next, 7 µL of piperidine solution was added, and the samples were incubated at 90°C for 30 min. After cooling, 20 µL of 13M ammonium acetate and 300 µL of cold (0°C) 96% ethyl alcohol were added, incubated for 30 min at −20°C and centrifuged for 10 min at 15,850 × g. The pellet was washed twice with 70% ethyl alcohol, dried under vacuum for 10 min, and then dissolved in 4 µL of loading buffer for sequencing gels. The samples were placed in a boiling water bath for 3 min, then cooled to 0°C and loaded into wells of the 8% sequencing gel. Electrophoresis was carried out in TBE buffer at 2000V, 95W and 55°C–60°C for 1.5–2 h.

### 2.9 Audioradiography and quantitative analysis

After the electrophoresis was completed, X-ray film and an intensifying screen were placed on the gel containing the radioactively labelled DNA, placed in an autoradiography cassette and exposed at −70°C for 1–2 h and 48 h for native gels containing complexes or sequential gels, respectively. Quantitative analysis of complexes were carried out using the computer program Gel Scan ver. 1.13, which enables quantitative analysis of the intensity of the bands recorded on the X-ray film during autoradiography.

### 2.10 Statistical analysis

The graphics and the data were constructed and analyzed statistically by means ± SD. Error bars are from three independent experiments.

## 3 Results

### 3.1 Obtaining DNA fragments containing the A1 promoter with an altered −35 region (fragment A1_−35_), an altered −10 region (fragment A1_−10_, A1_1/2–10_), and DNA fragments containing both changed −35 and −10 regions—fragments A1_−35/−10_ and A1_−35/1/2–10_

**FIGURE 1:**
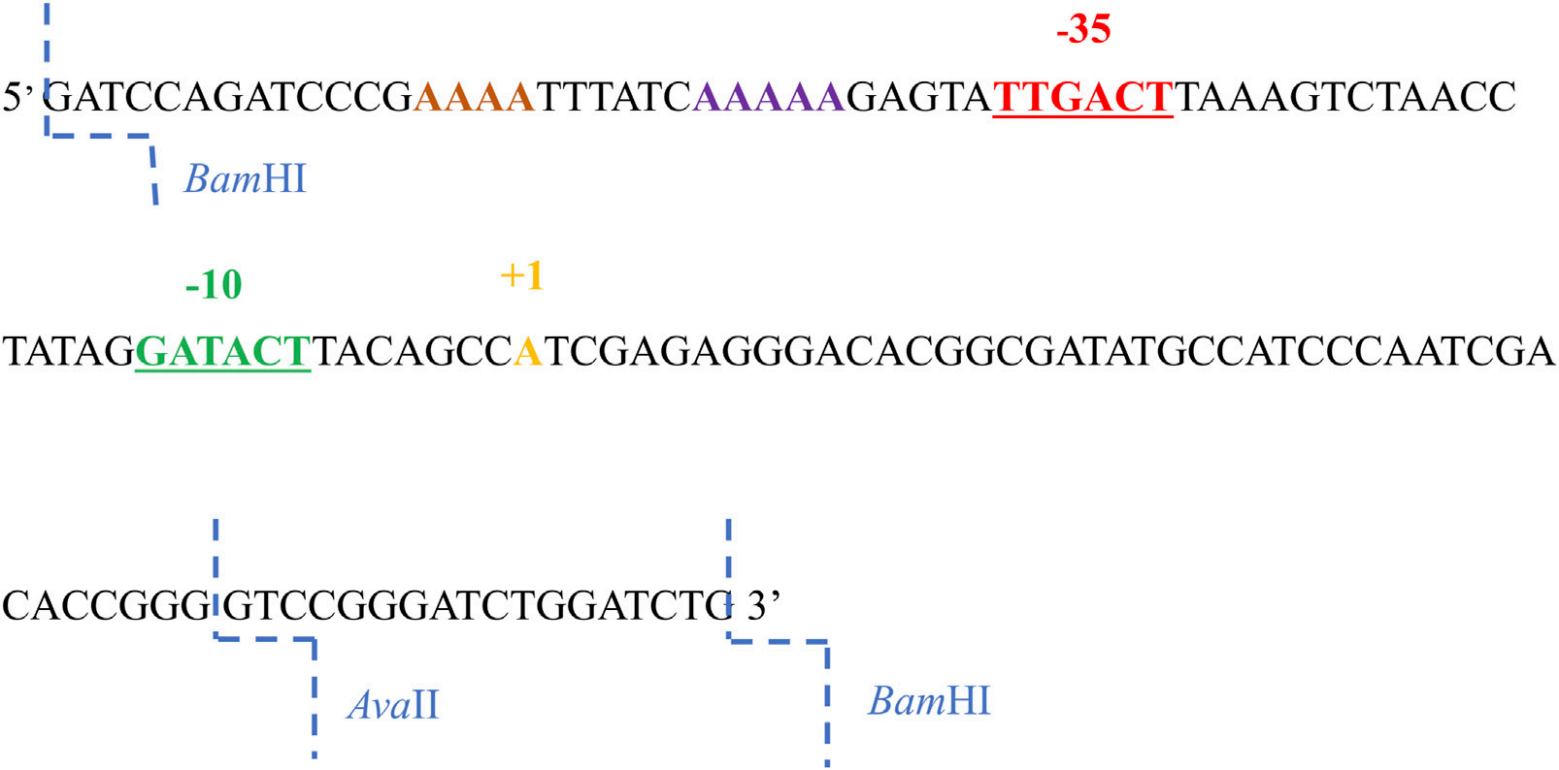
The 130 nucleotide sequence of the non-template strand of the promoter A1 of the phage T7. The −35 sequence is marked in red, the sequences in the −10 region in green, the transcription start site in yellow, AT-rich region in purple and the *Bam*HI and *Ava*II restriction enzyme cleavage sites in blue.

The A1 promoter sequence of phage T7 contains several sequences recognized by restriction enzymes; however, they are located downstream of the transcription start site (Figure 1). Because my interest was in the sequences located upstream of the transcription site, primarily the bases located within and near the −35 and −10 regions, additional restriction sites were introduced into the T7 phage A1 promoter, enabling modifications in the given sequences, as well as the analysis of RNA polymerase complexes with modified promoters containing both the −35 and −10 promoter regions altered (fragments A1_−35/−10_, and A1_−35/1/2−10_).

In order to analyze the effect of the −35 region sequence on RNA polymerase binding, a completely altered DNA sequence was introduced in this region of the A1 promoter of phage T7. The TTGACT sequence was replaced with the GAATTC sequence, which is recognized by the restriction enzyme *Eco*RI (non-template strand). The order of bases changed, while the order of bases of the number of hydrogen bonds, which determine the stability of this DNA segment, remained unchanged.

To analyze the influence of the −10 region of the promoter on the binding of RNA polymerase and the formation of the active initiation complex, knowing that the bases at the −12, −11, and −10 positions in the consensus promoter are of particular importance, two DNA fragments were constructed with a fully and partially altered sequence within the −10 hexamer. In the first fragment, the GATACT sequence of the A1 promoter was replaced with the ACAGAG sequence (fragment A1_−10_), while in the second fragment the GATACT sequence was replaced with the GATGAG sequence (fragment A1_1/2−10_).

**FIGURE 2:**
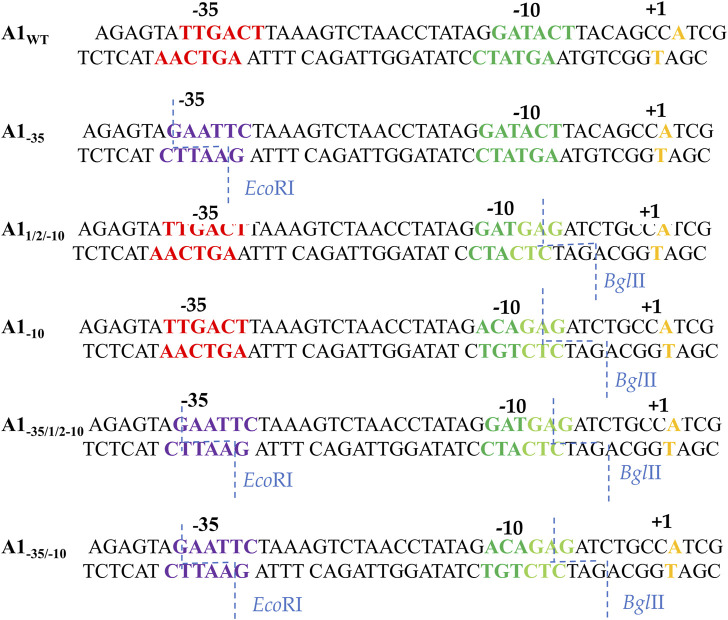
Sequences of the A1 promoter of the phage T7 fragments wild-type and modified in the −35 and −10 region. Red shows the −35 hexamer sequence, dark green—the unmodified −10 region sequence, light green—the new −10 hexamer sequence, purple—the new −35 hexamer sequence, orange—the transcription start site. The blue dashed line indicates the exact sites of *Bgl*II and *Eco*RI cleavage.

Thus, in the first case, the −10 hexamer was completely changed, while in the second, the bases corresponding to the −10, −9, and −8 positions were changed. By introducing changes in the −10 region, the bases were chosen to introduce a sequence recognized by the *Bgl*II enzyme, which allowed further modifications near the −10 region (Figure 2). Promoter DNA fragments with completely altered −35 and −10 regions and fully altered −35 region and a partially altered −10 region were also constructed (fragments A1_−35/−10_ and A1_−35/12/−10_) using, as a template, a promoter containing a modified hexamer–35 (A1_−35_) sequence. Changes were made in the −35 and −10 regions to correspond to the recognition sequences of the restriction enzymes *Eco*RI and *Bgl*II, respectively (Figure 2).

### 3.2 Interactions of A1_−35_, A1_−10_, A1_−1/2/−10_, A1_−35/−10_, and A1_−35/1/2−10_ fragments with *E. coli* RNA polymerase holoenzyme

#### 3.2.1 The interaction of modified fragments of DNA with the holoenzyme of RNA polymerase—the reaction of forming binary complexes

The modified promoter fragments of A1 of phage T7 and wild-type A1 fragment radioactively labelled at the 5′ end of one or both DNA strands were used to study the interaction of the RNA polymerase holoenzyme with the promoter DNA. In the labelling reaction, the T4 phage polynucleotide kinase enzyme and the ^32^P phosphorus isotope in the form of γ-labelled nucleoside triphosphate—[γ-^32^P]ATP were used.

Reaction conditions were optimized based on the unchanged A1 promoter of bacteriophage T7 radioactively labelled at the 5′ end of the template strand because the ranking of the −35 and −10 hexamers of the promoter fragments must be referenced to a standard, which in this case is the wild-type A1 promoter. In the further part of the research on the efficiency of the formation of binary complexes by modified promoter fragments, optimal concentrations of DNA and holoenzyme were used, as well as a holoenzyme with previously experimentally selected optimal concentrations of the core and sigma subunit. In each case, the complex formation reaction was carried out at 37°C in the presence of a low ionic strength buffer (8 mM Tris-HCl pH 7.9, 50 mM NaCl). Microdialysis on appropriate membrane filters (VS 0.025 μm Millipore) was used, in which the volume of the dialyzed sample may be as much as several microliters. After dialysis, heparin (at a concentration of about 1 mg/mL), which breaks up non-specific complexes, was added to the sample and applied to the gel. Optimal concentration of heparin (1 mg/mL) used in the creation of binary complexes experiments, was determined by testing a wide range of concentrations of this competitor (2.5 mg/mL-0.05 mg/mL). Only at the concentration of 1 mg/mL, a single band was visible, corresponding to the RNA polymerase holoenzyme complex with the promoter sequence (results not shown).

**FIGURE 3:**
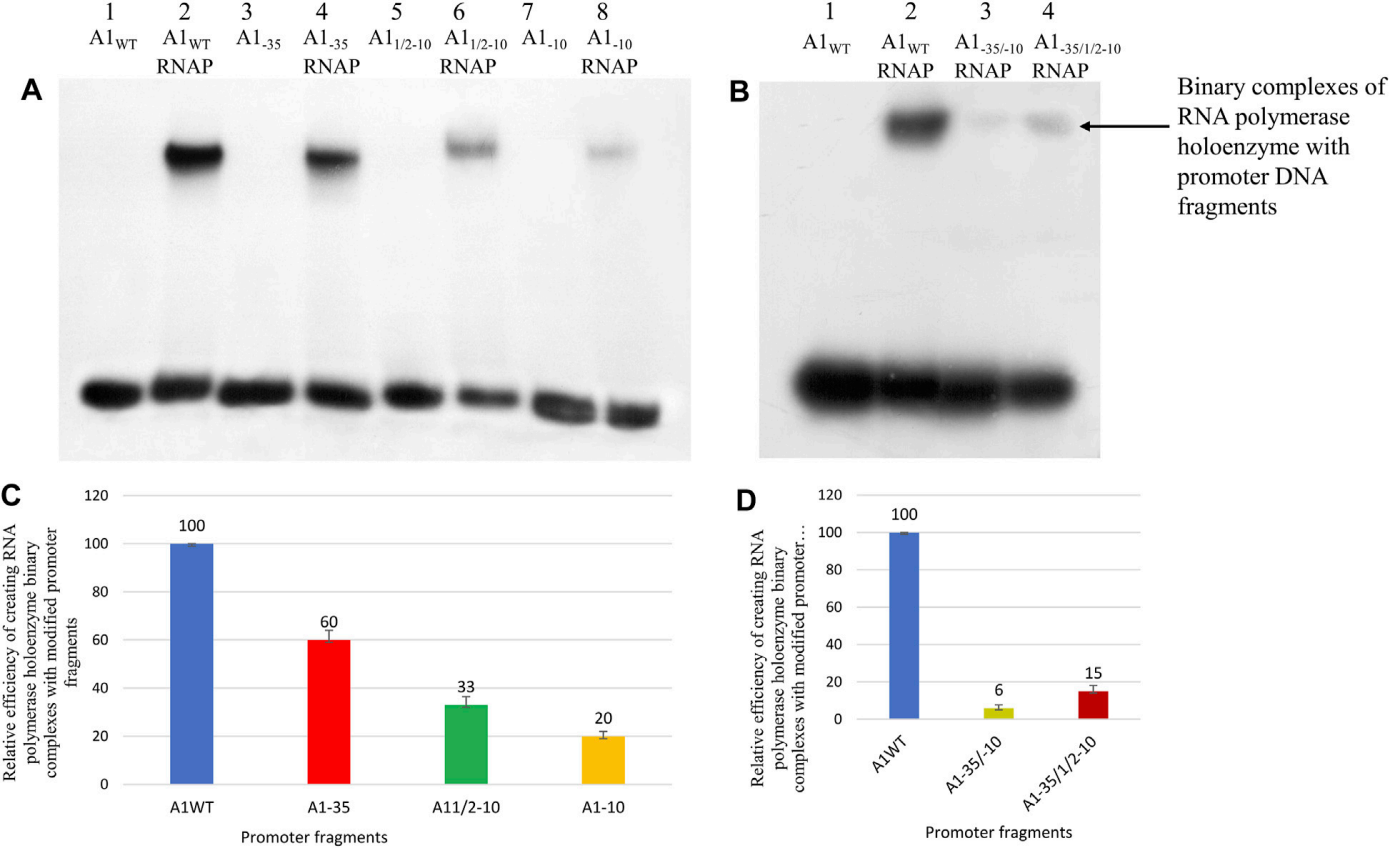
Analysis of RNA polymerase holoenzyme-promoter binary complexes formation; A1_WT_, A1_1/2−10_, A1_−10_, A1_−35_, A1_−35/−10_ and A1_−35/1/2−10_ labelled at the 5′ end of template DNA strands with ^32^P were used. In order to eliminate non-specific interactions, heparin with a concentration of 1 mg/mL was used. **(A,B)** Autoradiography of electrophoresis was carried out in a 3.5% polyacrylamide gel under non-denaturing conditions: **(A)** lane 1—A1_WT_ fragment; line 2—RNA polymerase holoenzyme binary complex with A1_WT_; lane 3—A1_−35_; line 4—RNA polymerase holoenzyme complex with fragment A1_−35_; lane 5—A1_1/2–10_; lane 6—RNA polymerase holoenzyme complex with fragment A1_1/2–10_; lane 7—A1_−10;_ line 8—RNA polymerase holoenzyme complex with fragment A1_−10_; **(B)** lane 1—A1_WT_; lane 2—RNA polymerase holoenzyme binary complex with fragment A1_WT_; line 3—RNA polymerase holoenzyme binary complex with fragment A1_−35/−10_; line 4—RNA polymerase holoenzyme binary complex with fragment A1_−35/1/2−10_. **(C)** Comparison of the relative efficiency of the RNA polymerase holoenzyme-the modified fragments **(C)** A1_−35_, A1_1/2−10_, A1_−10_, and **(D)** A1_−35/1/2−10_, A1_−35/−10_ binary complexes formation. Numbers above the bars indicate the percentage of the DNA-RNA polymerase complex formed relative to complex created by fragment A1 (100%). Data shown are mean ± SD. Error bars are from three independent experiments.

After determining the optimal conditions necessary for the formation of binary complexes in which the A1 promoter of the T7 phage was used, experiments were carried out to determine the effectiveness of the formation of RNA polymerase holoenzyme complexes with DNA fragments carrying the changed −10 and −35 regions. For the experiments devoted to the analysis of the formation of binary complexes, fragments of promoter DNA with the final concentration of 35 ng/μL were used radioactively labelled at the 5′ end. As in the case of the A1 promoter of the wild-type T7 phage, also in the case of the modified promoter fragments a DNA competitor was used: heparin with a starting concentration of 20 mg/mL (the final concentration in the reaction mixture was about 1 mg/mL). The efficiency of formation of binary complexes was checked by electrophoresis in a 3.5% polyacrylamide gel under non-denaturing conditions. The result of the experiment is illustrated in Figures 3A, B.

The obtained results show that in the case of the hexamers with completely altered sequences (A1_−35_, A1_−10_), bands corresponding to the complex of DNA with the RNA polymerase holoenzyme were obtained. Small amounts of binary complexes were also obtained for the fragments containing both hexamers altered simultaneously (A1_−35/1/2−10_, A1_−35/−10_). In the case of the A1_−35_ fragment, the band corresponding to the RNA polymerase holoenzyme complex with this fragment is less intense than the band corresponding to the RNAP complex with the A1 fragment, but the intensities of the bands indicate high reactivity of the fragment carrying the altered −35 region. On the other hand, complexes with fragments A1_1/2−10_ and A1_−10_ are much weaker than complexes with DNA fragments containing the A1 promoter, and even compared to the fragment containing the altered hexamer −35 (A1_−35_). The formation efficiency of all complexes was determined in relation to the activity of the DNA fragment containing the A1 promoter, which was assumed to be 100% (Figures 3C, D).

The results of the experiment show that the change of the TTGACT sequence to the GAATTC within the −35 region does not have a spectacular effect on the formation of the complex with the RNA polymerase holoenzyme compared to the unchanged fragment of promoter DNA. The promoter DNA fragment containing the modified −35 region exhibits about 55%–65% of the activity of the A1 promoter. Whereas, changes within the −10 region of the GATACT sequence to GATGAG (A1_1/2–10_) and ACAGAG (A1_−10_) significantly reduced the efficiency of complex formation by 67% and 80%, respectively. In fragments A1_−10_ and A1_1/2−10_ we observe the so-called additive effect of both parts of the −10 hexamer, because each of these parts contributes to complex stability. Changing the bases −9, −8, and −7 of the A1_1/2−10_ fragment reduced the efficiency of binary complex formation by about 70%, while the additional change of the second half of the hexamer reduced the efficiency by a further 13%. Also, in the case of fragments in which both −35 and −10 regions were changed, binary complexes were obtained, contrary to expectations, but with very low efficiency, corresponding to about 15% and 6%, respectively, of the activity of the A1 promoter of phage T7.

#### 3.2.2 Study of binary complexes with restriction enzymes

To explain the reasons for the different efficiency of the formation of binary complexes by promoter fragments modified in the −35 and −10 regions, the formed complexes were analyzed using restriction enzymes. Restriction enzymes were used to digest the tested DNA fragments in precisely defined locations, which are important for the formation of promoter complexes with the RNA polymerase holoenzyme (Figure 2). In these experiments, the formed binary complexes were digested in the presence of heparin with appropriate restriction enzymes: fragment A1_−35_ in complex with RNA polymerase holoenzyme was digested with *Eco*RI enzyme, complexes formed by fragments A1_−10_ and A1_1/2−10_ were digested with *Bgl*II enzyme, while complexes formed by double mutants A1_−35/−10_ and A1_−35/12−10_ were digested with *Eco*RI and *Bgl*II enzymes. The DNA fragments used in the experiment were labelled at both 5′ ends to independently analyze both fragments that may result from the digestion reaction. The digested complexes were separated from the free DNA by a filtration process on nitrocellulose membrane filters, as described in Section 2.7. The formed protein-DNA complexes were retained on the filter, while free DNA passed to the filtrate. The complexes immobilized on the filters were eluted, DNA was isolated from them and then analyzed by electrophoresis in a 6% polyacrylamide gel under non-denaturing conditions.

**FIGURE 4:**
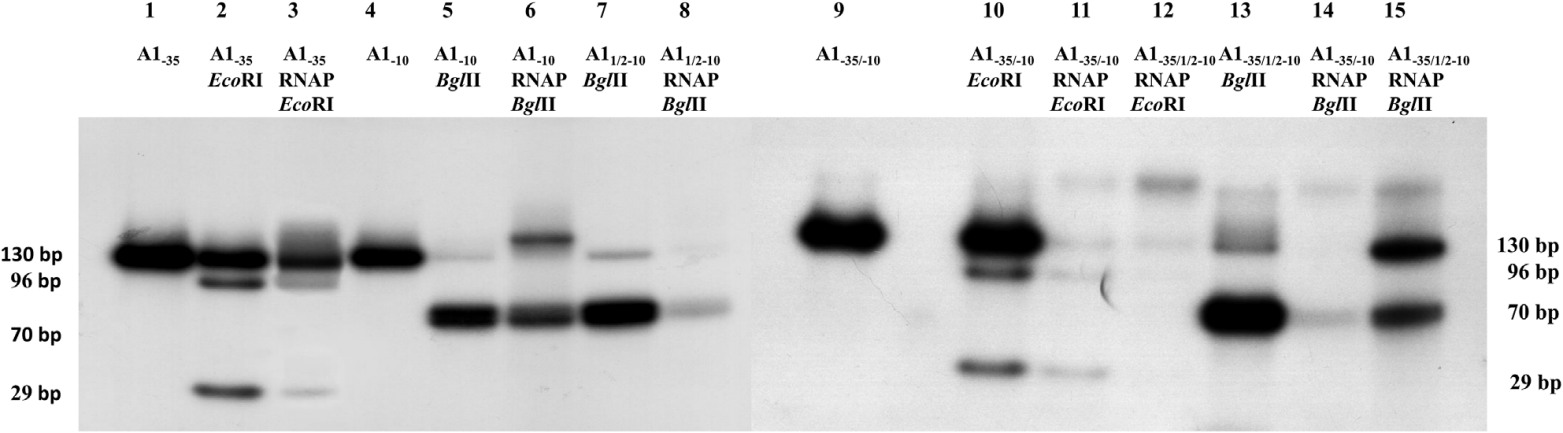
Autoradiography of RNA polymerase holoenzyme-fragments A1_−35_, A1_1/2−10_, A1_−10_, A1_−35/−10_ and A1_−35/1/2−10_ binary complexes restriction analysis. Electrophoresis was carried out in a 6% polyacrylamide gel under non-denaturing conditions: lane 1—fragment A1_−35_; lane 2—A1_−35_ fragment digested with *Eco*RI enzyme; lane 3—DNA isolated from the RNA polymerase holoenzyme-fragment A1_−35_ complex digested with *Eco*RI enzyme; line 4—fragment A1_−10_; lane 5—fragment A1_−10_ digested with *Bgl*II enzyme; lane 6—DNA isolated from the RNA polymerase holoenzyme-fragment A1_−10_ complex digested with the *Bgl*II enzyme; lane 7—fragment A1_1/2−10_ digested with *Bgl*II enzyme; lane 8—DNA isolated from the RNA polymerase holoenzyme-fragment A1_1/2−10_ complex digested with the *Bgl*II enzyme; line 9—fragment A1_−35/−10_; lane 10—A1_−35/−10_ fragment digested with *Eco*RI enzyme; lane 11—DNA isolated from the RNA polymerase holoenzyme-fragment A1_−35/−10_ complex digested with *Eco*RI; lane 12—DNA isolated from the RNA polymerase holoenzyme-fragment A1_−35/1/2–10_ complex digested with *Eco*RI; lane 13—fragment A1_−35/1/2−10_ digested with *Bgl*II enzyme; lane 14—DNA isolated from the RNA polymerase holoenzyme-fragment A1_−35/−10_ complex digested with the *Bgl*II enzyme; lane 15—DNA isolated from the RNA polymerase holoenzyme-fragment A1_−35/1/2−10_ complex digested with the enzyme *Bgl*II.

The control for these experiments was *Eco*RI and *Bgl*II digestion of free DNA (fragments A1_−35_, A1_−10_, A1_1/2−10_, A1_−35/−10_, and A1_−35/1/2−10_). This control was carried out in the presence of heparin to exclude the influence of this competitor on enzyme activity restriction. The results of electrophoretic analyses of DNA obtained from binary complexes treated with *Eco*RI and *Bgl*II restriction enzymes are presented in Figure 4.

Digestion of the modified promoter DNA fragments in a binary complex with the RNA polymerase holoenzyme proved to be successful in each case. In Figure 4, lanes 3, 11, and 12 two DNA bands of 96 bp and 29 bp are shown resulting from *Eco*RI cleavage of fragments A1_−35_ and A1_−35/−10_. These fragments, corresponding to the cleavage of free DNA, are also seen in lane 2. These results indicate that DNA containing the modified hexamer −35 in complexes with RNA polymerase is accessible to the restriction enzyme. This means that in this case there is no direct, intimate contact between the modified hexamer −35 and the RNA polymerase. Also, fragment A1_−10_ (completely altered hexamer −10) and A1_1/2−10_ as well as fragments A_−35/−10_ and A1_−35/1/2−10_ in the complex are affected by the restriction enzyme *Bgl*II, resulting in a fragment DNA corresponding to a length of about 70 bp (Figure 4, lanes 6, 8, 14, and 15). In this case, it cannot be unequivocally determined whether two fragments of DNA, which are the product of digestion with the *Bgl*II enzyme, or only one fragment are bound in the complex with the RNA polymerase. DNA cleavage by the restriction enzyme *Bgl*II in binary complexes proves that the reason for the low efficiency of complex formation observed in the case of the modified A_−10_ and A1_1/2−10_ promoters is the very weak interaction of the −10 region with the RNA polymerase (σ subunit of the polymerase). As a result, the enzyme cannot stably bind the sequence located in the −10 region, so a satisfactory amount of heparin-stable binary complex cannot be formed (Figure 3A).

#### 3.2.3 Investigation of interactions of the DNA sequence located upstream of the −35 hexamer on RNA polymerase binding

**FIGURE 5:**
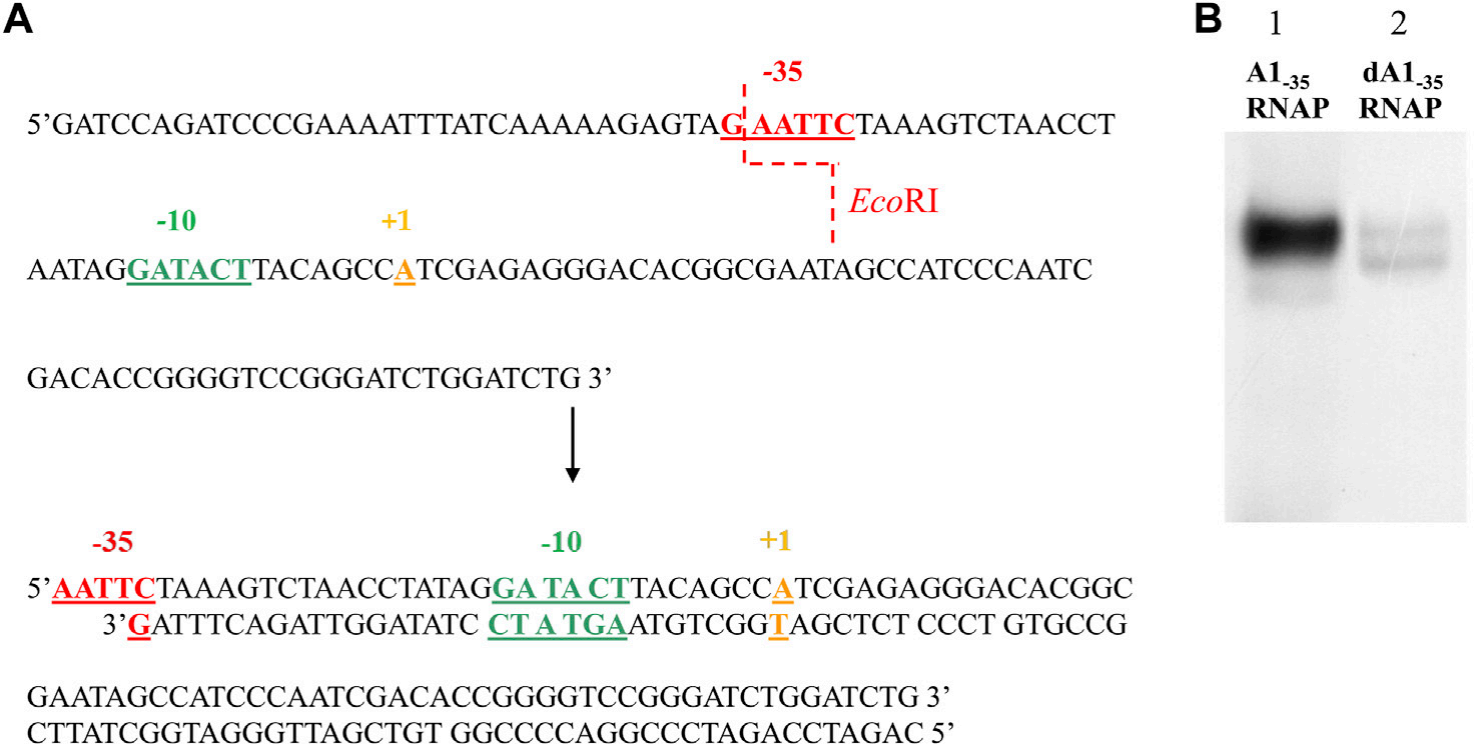
The result of digestion of the A1_−35_ fragment. Scheme of cleavage of the A1_−35_ fragment (non-template strand of the DNA fragment containing the altered −35 region) with the *Eco*RI enzyme and a double-strand cut product **(A)**. Autoradiography of the analysis of RNA polymerase holoenzyme-DNA fragments binary complexes: A1_−35_ and dA1_−35_ (fragment obtained as a result of A1_−35_ cleavage with *Eco*RI restriction enzyme) **(B)**. The complexes were formed in the presence of 1 mg/mL heparin. Electrophoresis was carried out in a 3.5% polyacrylamide gel under non-denaturing conditions: lane 1—RNA polymerase holoenzyme complex with A1_−35_ fragment, lane 2—RNA polymerase holoenzyme-dA1_−35_ fragment complex. The results were obtained in three independent experiments.

To analyze the impact of interactions of the DNA sequence located upstream of the −35 hexamer on the binding of RNA polymerase, the A1_−35_ fragment was used (the −35 region had a restriction site for the *Eco*RI enzyme). This enzyme cuts the DNA within the −35 hexamer and removes the sequence located above the −35 region. This should lead to the weakening of interactions with upstream sequences and reduce the efficiency of enzyme-DNA complex formation. To implement this assumption, the A1_−35_ fragment, containing the changed–35 region, was digested with *Eco*RI, generating the DNA fragment shown in Figure 5. The obtained fragment was named dA1_−35_ (Figure 5A). This fragment is much less effective in forming binary complexes with the RNA polymerase holoenzyme (Figure 5B). This suggests that the interactions responsible for the high efficiency of binary complex formation by the fragment with the changed hexamer −35 (A1_−35_) are located above of the −35 hexamer (UP element) and not in the downstream part of the promoter.

**FIGURE 6:**
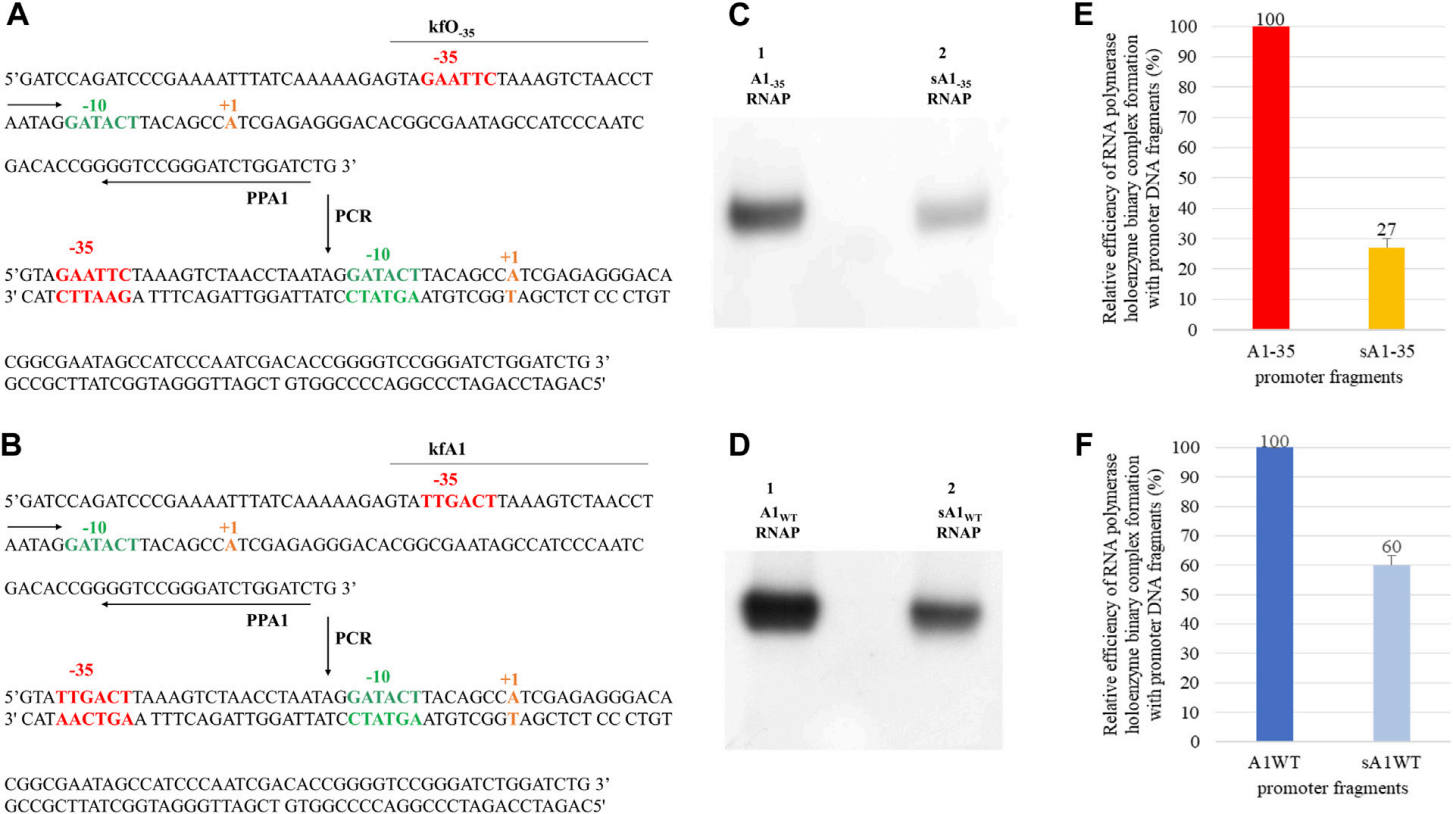
Method for obtaining **(A)** the A1 promoter of the phage T7 fragment containing an altered −35 region - lacking the sequence upstream of the −35 hexamer (upstream of the −39 position) and a wild type **(B)** A1 T7 promoter fragment; kfO_−35_, kfA1 and PPA1 are the primers used in the PCR reaction. Below are double-stranded PCR products - fragment sA1_−35_ and sA1_WT_. Autoradiography showing the analysis of the effectiveness of the RNA polymerase holoenzyme-DNA fragments binary complexes formation: **(C)** A1_−35_ (A1 promoter of the phage T7 containing a completely changed −35 region), sA1_−35_ (A1_−35_ fragment lacking the sequence located above the bases at position −39, obtained by PCR reaction) and A1_WT_; **(D)** sA1 (A1 promoter of the T7 phage lacking the upstream sequence at position −39), labelled with ^32^P at the 5′ end of the template strand. In order to eliminate non-specific interactions, heparin with a concentration of 1 mg/mL was added. Electrophoresis was carried out in a 3.5% polyacrylamide gel under non-denaturing conditions: **(C)** lane 1—RNA polymerase holoenzyme-A1_−35_ fragment binary complex, lane 2—RNA polymerase holoenzyme-sA1_−35_ fragment binary complex; **(D)** lane 1—RNA polymerase holoenzyme-A1 binary complex, lane 2—RNA polymerase holoenzyme-sA1 fragment binary complex. Comparison of the relative efficiency of the RNA polymerase holoenzyme-DNA (A1_WT_, sA1 and A1_−35_, sA1_−35_) complexes formation. The numbers above the bars indicate the percentage of the DNA-RNA polymerase complex with respect to complex formed by **(E)** A1_−35_ or **(F)** A1_WT_. Data shown are mean ± SD. Error bars are from three independent experiments.

To exclude the possibility that the weakening of the interactions of the RNA polymerase with the dA1_−35_ (digested A1_−35_) fragment obtained as a result of the *Eco*RI restriction enzyme (Figure 5B) is not the result of cleavage of the sequence located above the −35 region, but the result of impaired function of the −35 hexamer (a fragment shorter by one base in strandand partially single-stranded), an experiment was performed with a completely double-stranded DNA fragment. This fragment (sA1_−35_, short A1_−35_) contains an unbroken, double-stranded −35 hexamer and 3 bp upstream of this hexamer. It was obtained by PCR with primers kfO_-35_ and PPA1 (Figure 6A). This fragment was used for the formation of binary complexes with the RNA polymerase holoenzyme and was found to have activity similar, in interactions with RNA polymerase, to the partially single-stranded fragment in the −35 region, obtained by the action of the restriction enzyme *Eco*RI (Figure 6C).

This clearly shows that for the formation of complexes with RNA polymerase, it does not matter whether the truncated fragment, in the case of the modified −35 hexamer, is double-stranded, single-stranded, shorter, or longer by 3 bp. These experiments lead to the suggestion that also in the wild-type A1 promoter of phage T7, sequences located upstream of the −35 region make a significant contribution to the formation of binary complexes. To experimentally verify this suggestion, a shortened fragment of the A1 promoter wild type (sA1_WT_) of the same length as the sA1_−35_ fragment was obtained by PCR, using primers kfA1 and PPA (Figure 6B). This fragment then was used to analyze the formation of complexes with RNA polymerase. The result of the experiment is shown in Figure 6D. Also in this case, complexes with RNA polymerase are formed with a lower efficiency, which is about 60% of the activity of the 130 bp wild-type A1 promoter. Quantitative analysis of the complexes shown in Figures 6C, D was also carried out, as presented in Figures 6E, F.

Figures 6E, F shows that removal of the sequence located upstream of the −35 hexamer results in a significant reduction in the complexing efficiency of both the wild-type promoter and the promoter with the modified −35 sequence. In the latter case, however, the effect is much stronger, accounting for about 73% of the activity of the full-length DNA fragment (A1_−35_). It also means that the decrease in the efficiency of the formation of binary complexes in relation to the wild-type promoter drops to a value close to 10%, i.e., to the level of non-specific DNA-protein interactions. This also demonstrates the additive effect of the sequence located upstream of the −35 region and the −35 hexamer in the formation of binary complexes. By shortening the DNA fragment containing the wild-type A1 promoter, the unchanged hexamer −35 present in the promoter can still perform its function in stabilizing the complex. The A1_−35_ fragment lacks the −35 hexamer; therefore, removing the sequence upstream of this promoter region eliminates the ability to form binary complexes to a greater extent.

### 3.3 Study of the type of binary complex created by the A1 promoter of the T7 phage containing the changed region −35 (A1_−35_) with the *E. coli* RNA polymerase holoenzyme

**FIGURE 7:**
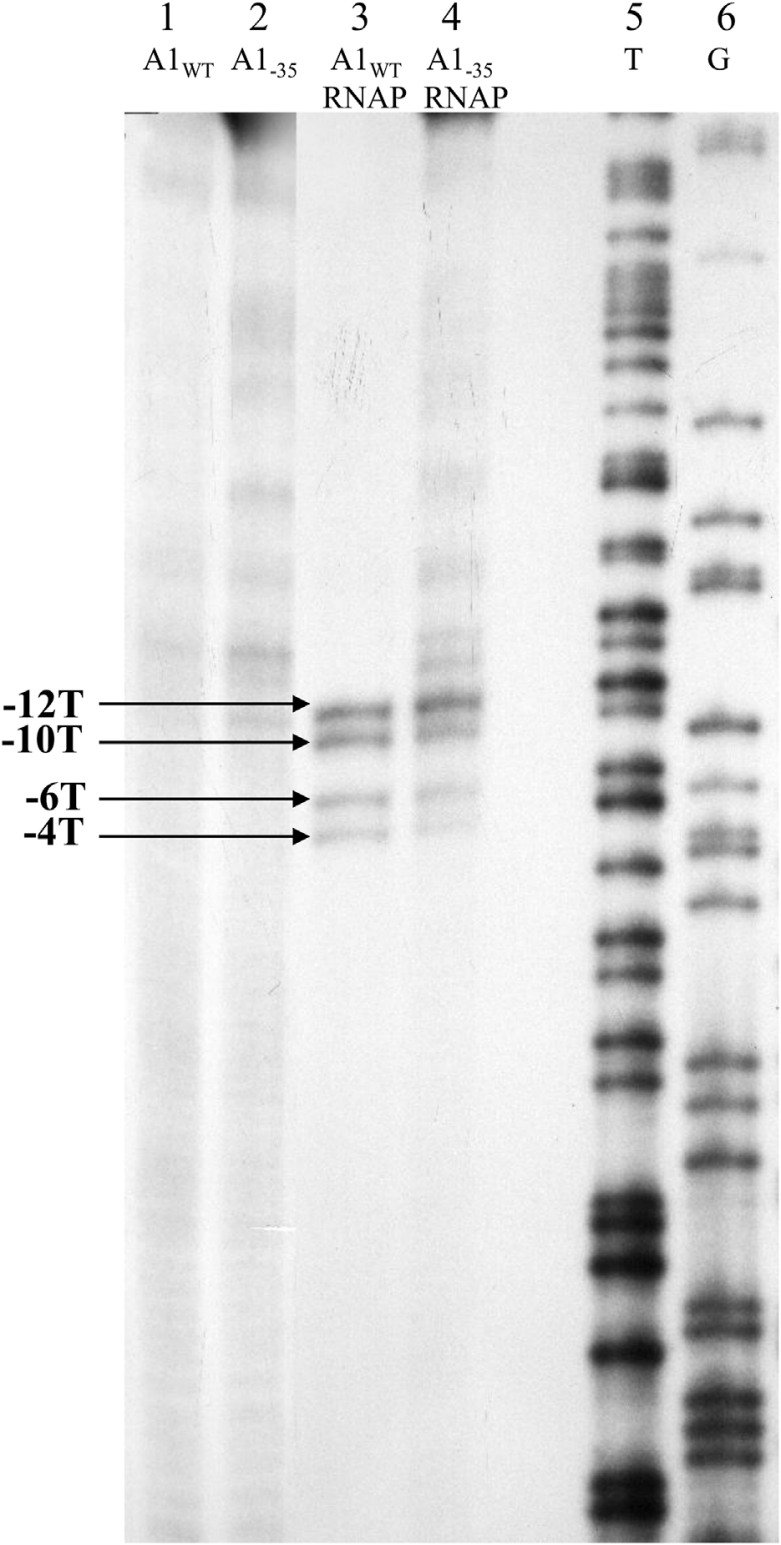
Autoradiography of the experiment assessing the availability of thymines of the DNA template strand in the binary complex using 1.5 mM KMnO_4_. Electrophoresis was carried out in an 8% sequential gel under denaturing conditions: lane 1—fragment A1 of phage T7 modified with KMnO_4_; lane 2—A1_−35_ fragment modified with KMnO_4_; lane 3—A1_WT_ promoter of the phage T7 in complex with RNA polymerase holoenzyme modified with KMnO_4_; lane 4—A1_−35_ fragment in complex with RNA polymerase holoenzyme modified with KMnO_4_; lane 5—template T obtained by sequencing reaction of the A1 promoter of phage T7 (template strand); lane 6—pattern G obtained by sequencing reaction of the A1 promoter of phage T7 (template strand); −12, −10, −6 and −4 represent the positions of the thymines of the template strand available to KMnO_4_ in the open complex. The results were obtained in three independent experiments.

To determine whether the DNA in the promoter fragment complex containing the completely altered −35 region is single-stranded and the thymines of the −10 region show increased reactivity towards KMnO_4_, the RNA polymerase complex with the A1_−35_ fragment formed at 37°C was modified with potassium permanganate. Complexes of RNA polymerase holoenzyme with wild-type T7 A1 promoter were used as controls. Modified DNA fragments isolated from the complex were analyzed on an 8% sequencing gel. The results of the experiments are presented in Figure 7.

The results of experiments using potassium permanganate show that both in the case of complexes of the RNA polymerase holoenzyme with the A1 promoter of T7 phage containing the completely changed region −35 and the wild-type A1 promoter of T7 phage, thymine at positions −12, −10, −6, and −4 are modified by potassium permanganate. In contrast, controls using free double-stranded promoter DNA fragments (A1, A1_−35_) showed no reactivity of thymine bases at these positions. These results are direct evidence that the complexes of the T7 phage wild-type A1 promoter and the completely altered −35 region with the RNA polymerase holoenzyme are open complexes.

### 3.4 Study of the activity of the initiation complex in the transcription process

For a more complete functional analysis of the obtained constructs, *in vitro* transcription experiments were performed to determine whether the complexes obtained as a result of binding of the RNA polymerase holoenzyme to the changed promoters (A1_−10_, A1_1/2−10_, A1_−35_, A1_−35/−10_, A1_−35/1/2−10_) are able to enter the productive phase of transcription and RNA synthesis.

Thus, the transcription reaction was performed using the ApUpC primer and 2 of the 4 ribonucleotides (ATP and GTP). Under these conditions, an RNA product of 11 nucleotides is formed. This is due to the DNA sequence of the T7 phage A1 promoter, which is in the strand non-template from +1 (transcription start site) to +12, which contains the following nucleotides: 5′-ATCGAGAGGGAC-3′. Therefore, by carrying out transcription reactions under substrate-limiting conditions, we obtain the RNA of 11 nucleotides. The resulting ternary complex is characterized by slightly greater electrophoretic mobility than the binary complex. The increase in the mobility of the ternary complex in relation to the binary complexes is associated, on the one hand, with the removal of the sigma subunit from the holoenzyme and, on the other hand, with the increase of the negative charge given to the complex by the RNA product of the reaction. The resulting triple complex is visible on the gel thanks to the DNA labelled at the 5′ end, and the RNA product thanks to the radioactively labelled ApUpC primer.

**FIGURE 8:**
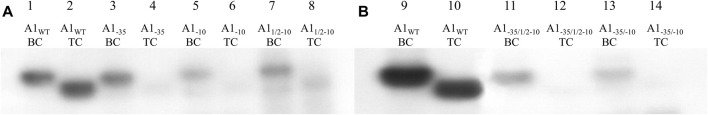
Autoradiography of the electrophoretic analysis of RNA polymerase holoenzyme-fragments A1_WT_, A1_−35_, A1_−10_, A1_1/2–10_, A1_−35/1/2–10_ and A1_−35/−10_ binary and ternary complexes; DNA fragments were labelled at the 5′ end of template strand with radioactive ^32^P. Electrophoresis was carried out in a 3.5% polyacrylamide gel under non-denaturing conditions: **(A)** lane 1—RNA polymerase holoenzyme-fragment A1_WT_ binary complex (BC), lane 2—RNA polymerase holoenzyme-fragment A1_WT_ ternary complex (TC), lane 3—RNA polymerase holoenzyme-fragment A1_−35_ binary complex (BC), lane 4—RNA polymerase holoenzyme A1_−35_ fragment ternary complex (TC), lane 5—RNA polymerase holoenzyme-A1_−10_ fragment binary complex (BC), lane 6—RNA polymerase holoenzyme-A1_−10_ fragment ternary complex (TC), lane 7—RNA polymerase holoenzyme-A1_1/2−10_ fragment binary complex (BC), lane 8—RNA polymerase holoenzyme-A1_1/2−10_ fragment ternary complex (TC); **(B)** lane 9—RNA polymerase holoenzyme-fragment A1_WT_ binary complex (BC), lane 10—RNA polymerase holoenzyme-fragment A1_WT_ ternary complex (TC), lane 11—RNA polymerase holoenzyme-fragment A1_−35/1/2−10_ binary complex (BC), lane 12—RNA polymerase holoenzyme-fragment A1_−35/1/2−10_ ternary complex (TC), lane 13—RNA polymerase-fragment A1_−35/−10_ binary complex (BC), lane 14—ternary complex of RNA polymerase holoenzyme-fragment A1_−35/−10_ ternary complex (TC). The results were obtained in three independent experiments.

Six binary complexes obtained with fragments A1, A1_−10_, A1_1/2−10_, A1_−35_, A1_−35/−10_, and A1_−35/1/2−10_ were used in a limited transcription reaction. The control was a DNA fragment containing the intact A1 promoter of phage T7. Reaction is presented in Figure 8.

The results of the *in vitro* transcription reaction under conditions of limited substrates (for radioactively labelled DNA fragments) analyzed in polyacrylamide gels under non-denaturing conditions showed, in all cases of modified promoters, only trace amounts of radioactivity in the position corresponding to the positions of the triple complexes, with significantly higher electrophoretic mobility (Figure 8, lanes 4, 6, 8) compared to bands of binary complexes (Figure 8, lanes 3, 5, 7). Bands corresponding to triple complexes are better seen with long X-ray exposure times. Such bands corresponding to the positions of the triple complexes, although of very low intensity, were also formed in the case of fragments containing completely and partially changed region −10. On the other hand, the fragments containing both altered hexamers at the same time (A1_−35/−10_ and A1_−35/1/2−10_) show practically no blackening of the film in this position. All complexes with higher electrophoretic mobility corresponding to the modified promoters are much less intense than the triple complex with the A1 fragment.

**FIGURE 9:**
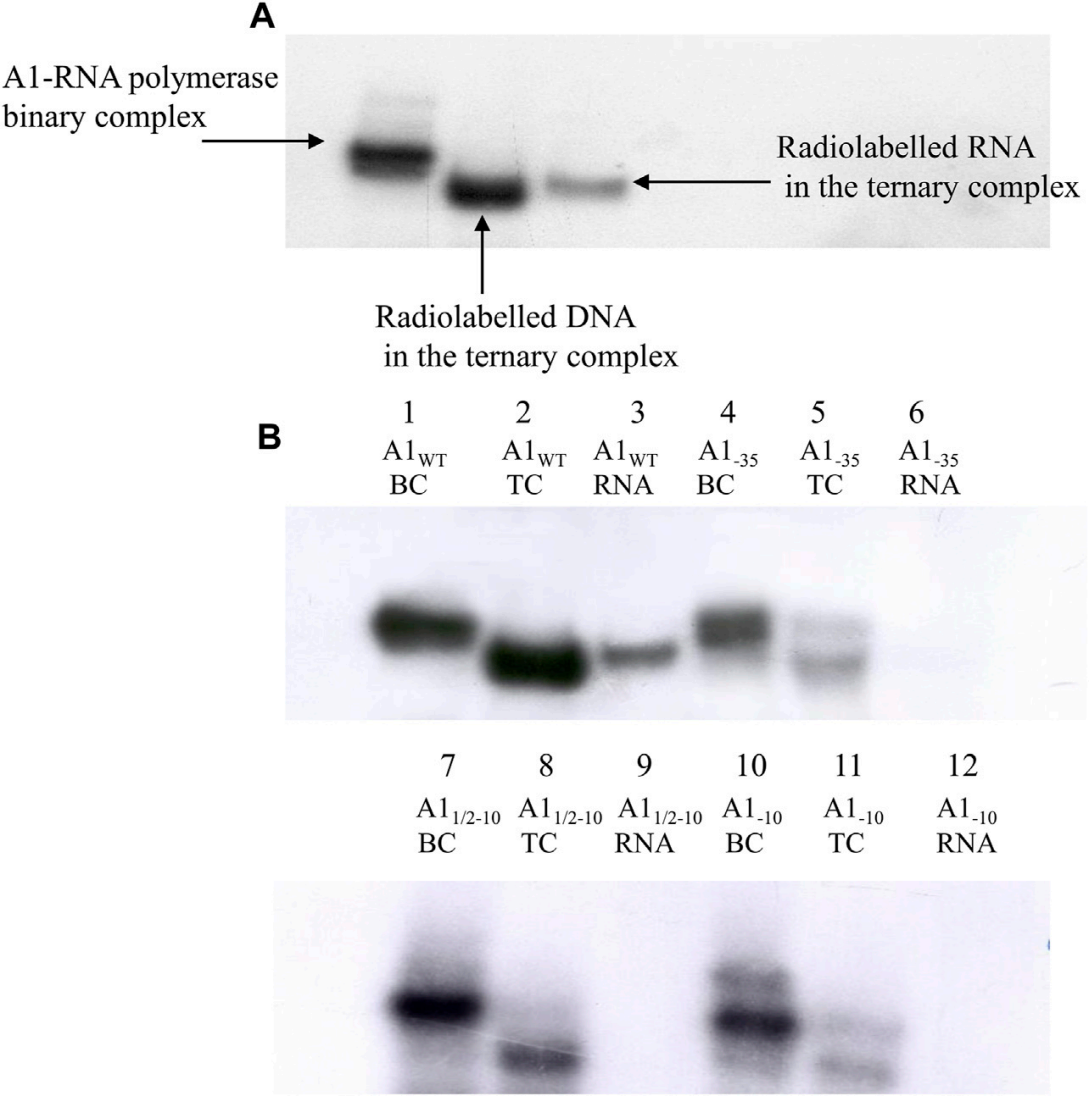
Autoradiography of the *in vitro* transcription reaction results analyzed on the 3.5% polyacrylamide gel under non-denaturing conditions. **(A)** RNA polymerase holoenzyme-A1 promoter of the phage T7 binary and ternary complexes; a radioactively labelled primer ApUpC was used, resulting in a labelled RNA product, or a labelled DNA fragment containing the A1_WT_ promoter of phage T7. **(B)** Autoradiography showing analysis of the *in vitro* transcription reaction using labelled RNA product; lanes 1, 4, 7, 10—RNA polymerase holoenzyme-A1_WT_, A1_−35_, A1_1/2−10_, A1_−10_ fragments binary complexes (BC), respectively (labelled with radioactive ^32^P at the 5′ end of the template strand), lanes 2, 5, 8, 11—RNA polymerase holoenzyme-fragments A1_WT_, A1_−35_, A1_1/2−10_, A1_−10_ ternary complexes (TC), respectively, lane 3—RNA product in a ternary complex formed by RNA polymerase with the A1_WT_ promoter of phage T7 (A1_WT_ RNA); lane 6—traces of RNA product (A1_−35_ RNA), lanes 9, 12—no RNA product (A1_1/2−10_ RNA, A1_−10_ RNA, respectively). The image shows the autoradiogram of the gel subjected to a very long exposure time. The results were obtained in three independent experiments.

The ability of the modified promoter fragments to carry out the *in vitro* transcription reaction was further verified in experiments in which the radioactively labelled primer ApUpC was used, tagged transcription product. The results of these experiments showed that the transcriptionally active complex is only the RNA polymerase holoenzyme complex with the unaltered A1 promoter fragment of T7 phage (Figure 9A). In the case of the DNA fragment containing the altered −35 region, a faint band corresponding to the RNA product was observed only after long X-ray exposure. However, due to its low intensity, this fringe is not visible in the image (Figure 9B, line 6). On the other hand, in experiments with DNA fragments containing a completely and partially altered region −10, no RNA product was found (Figure 9B), even with a very long X-ray exposure (no blackening of the film in lines 9 and 12). In the case of the A1 promoter of phage T7 containing a partially altered hexamer −10, some radioactivity from the binary complex appears at the position of the ternary complex (visible on long exposure in Figure 9B, lane 8), which would suggest that the binary complex is able to pass through, with low efficiency, to the productive phase of transcription. However, the absence of a labelled RNA product (lane 9) does not conclusively indicate that transcription is successful in this case. It also appears that in both cases of promoters altered in the −10 region (A1_1/2−10_ and A1_−10_) the binary complexes become heparin sensitive when transcription is attempted.

## 4 Discussion

The critical moment in the transcription process is the initiation step, in which the RNA polymerase holoenzyme (*E. coli* promoters) specifically recognizes two evolutionarily conserved regions: −35 and −10 (Bown et al., 1999). In addition to the specific interactions with these hexamers, there are several non-specific interactions, among them relatively strong ones binding RNA polymerase to the ends of the molecule in a linear form, but also binding to any other random sequence within the DNA. These non-specific interactions make it easier for RNA polymerase to locate promoter sequences more rapidly in the early stages of transcription initiation (von Hippel. et al., 1984). It is already known that the −35 and −10 regions play an important role in transcription initiation process and interact with *E. coli* RNA polymerase (Siebenlist and Gilbert, 1980; Chenchick et al., 1981; Campbell et al., 2002; Feklistov and Darst, 2011; Campagne et al., 2014; Feklistov et al., 2014). Also, any manipulation within the sequences of these hexamers results in changes in affinity enzyme to the promoter, and it affects the promoter strength (Siebenlist and Gilbert, 1980; Youderian et al., 1982; Urtecho et al., 2019). In hydroxyl radical interference experiments, where free hydroxyl radicals act on double-stranded promoter DNA (radicals act on the DNA molecule by randomly removing a single nucleoside from one strand, thus damaging the DNA and eliminating a possible contact point with RNA polymerase) (Tullius and Dombroski, 1986; Hayes and Tullius, 1989; Shafer et al., 1989), it was found that nucleoside deletions analyzed in the broad context of the promoter sequence and the unique thermodynamic conditions of complex formation produce a location-dependent effect of such a deletion. It was shown also that the deletions prevent the formation of binary complexes under these conditions, and thus limit all other phases of transcription initiation, and they are located in the −35 region of the template strand. At the same time, deletions of this type located in the −10 region did not have such a negative effect on binding. The experiment was carried out at a temperature of 4°C, at which the RNA polymerase does not form a stable open complex with the native DNA promoter. The increased affinity of the enzyme for DNA in this region is therefore due to the increased torsion flexibility of the DNA molecule due to the deletion of a single nucleoside, which probably allows the DNA to assume an appropriate, favorable conformation and form a stable open complex, even at 4°C (Werel et al., 1991). We pose a hypothesis, that both the negative and positive nucleoside deletion effects observed for different regions of the promoter DNA are the result of anisotropic DNA bending resulting from the deletion (Guo and Tullius, 2003).

Analysis of DNA fragments containing altered −35 and −10 hexamers for their ability to form binary complexes showed that these promoter fragments have reduced affinity for RNA polymerase relative to the unaltered A1 promoter of phage T7. However, this effect varies depending on which hexamers have been modified and what type of changes have been made to the hexamer sequence. There seems to be a complete change in the DNA sequence in the −35 region, which does not have as much influence on the interaction with RNA polymerase as a change in the −10 region. Experiments show that even a partial change in the hexamer −10 (altered bases −10, −9, −8) with the “upper” part of this region unchanged (bases −13, −12, and −11) causes a significant weakening of the interaction until about 70%. All experiments were carried out in the presence of heparin. This compound is a polyanion, and it competes with DNA for the binding site with RNA polymerase by attaching to the β′ subunit and thus weakens the non-specific binding of the enzyme, e.g., to the ends of the DNA or to random sequences within the DNA. Thus, the complexes formed under these conditions are stable, in the form of intermediate or open complexes. A special feature of the open complex is its resistance to high concentrations of heparin, which means that the RNA polymerase must be correctly located in the promoter sequence. Experiments with the altered −35 region (Figure 3A) have shown that even a complete alteration of the −35 sequence is not very significant for the formation of binary complexes. A complete replacement of the promoter sequence of the −35 region results in a reduction of the efficiency of complex formation by about 35%–45%. Niedziela-Majka and Heyduk (2005) suggested that the −35 site is not essential for open complex (RPo) formation, and strand separation can still occur in the complete absence of this motif (Niedziela-Majka and Heyduk, 2005). In turn, Urtecho et al. (2019) using a promoter library showed that the most active promoters (from which expression was the highest) were when one, but not both, elements matched the consensus (Urtechto et al., 2019). In our work, it is also important that by modifying hexamer −35, its sequence was changed, but the total number of hydrogen bonds remained unchanged. From an energetic point of view, the sequences of the −35 region in the unaltered A1 promoter and the altered A1_−35_ fragment are equivalent. This weak modification effect thus supports the suggestions from interference analyses that deletion of a nucleoside in the −35 region of the non-template strand has no negative effect on RNA polymerase binding, regardless of the position of the deleted nucleoside. The lack of a clear relationship between the effect of the deletion and its location, in the case of the template strand, points to pure physical effects on the formation of a binary complex with DNA treated with hydroxyl radicals, not the attenuation of specific protein-DNA interactions. This suggests that a significant contribution to the stability of the complex is made by UP element-polymerase interactions, including also non-specific interactions of the −35 region. Von Hippel (2007) and Rhodius and Mutalik (2009) in theirs works presented that the loss of specific protein-DNA interactions results in abrupt transition nonspecific electrostatic interactions (von Hippel, 2007; Rhodius and Mutalik, 2009). In our work it is also strongly supported by the fact that the shortened DNA fragments are deprived of sequences above the −35 region, obtained by PCR or by cleavage with the *Eco*RI enzyme (fragments sA1_−35_ and dA1_−35_, respectively), and show much lower RNA polymerase binding capacity (about 10%–15%) (Figures 5B, 6C, 7B). This is probably due to the attenuation of bindings contributed by the part of the promoter located upstream of the −35 region. Also, in the case of the truncated DNA fragment containing the promoter A1 of the wild-type phage T7 (sA1), a decrease in the efficiency of binary complex formation was observed (Figures 6D, 7A). This suggests the additive contribution of the sequence located upstream of the −35 hexamer and the −35 region in interaction with RNA polymerase to form binary complexes. The relatively high efficiency (about 60%) of binary complex formation in the case of the truncated wild-type A1 promoter of phage T7 proves that the unmodified hexamer −35 still contributes to the stabilization of such a complex. On the other hand, in the case of the A1_−35_ fragment containing the completely altered −35 region, deletion of the whole of the sequence upstream of this hexamer causes a drastic decrease in activity (about 10%–15% of the activity of the wild-type A1 promoter), caused by a lack of close contact with the RNA polymerase of both the sequence located upstream of the −35 region and the hexamer. Models of binary complexes of RNA polymerase with promoter DNA assume that the DNA in the complex is wrapped around the protein (wrapping), making a significant contribution to the stabilization of the complex by additional contact with RNA polymerase, located upstream of the −35 region (Craig et al., 1990; Rivetti et al., 1999; Travers, 2015). It should be emphasized also that regardless of whether the shortened DNA fragment with the changed −35 region contained a completely double-stranded −35 hexamer (a product of the PCR reaction) or mostly single-stranded (the effect of cleavage with the restriction enzyme *Eco*RI), the efficiency of binary complex formation was similar (Figures 5B, 6C). This suggests that the RNA polymerase is not in close contact with the modified −35 region. This is obvious considering the experiments devoted to the action of the restriction enzyme *Eco*RI on binary complexes of RNA polymerase with fragments containing a modified hexamer −35. In this study it has been shown that the sequence is accessible to the *Eco*RI enzyme, suggesting that there is no direct contact between the enzyme and the modified hexamer. Such close contact between the RNA polymerase and the −35 hexamer takes place in the *Salmonella typhimurium trp* promoter, which contains a naturally occurring restriction site for the *Hinc*II enzyme in the −35 region. In this case, in the open complex, this site is not accessible to the restriction enzyme (Oppenheim and Yanofsky, 1989).

**FIGURE 10:**
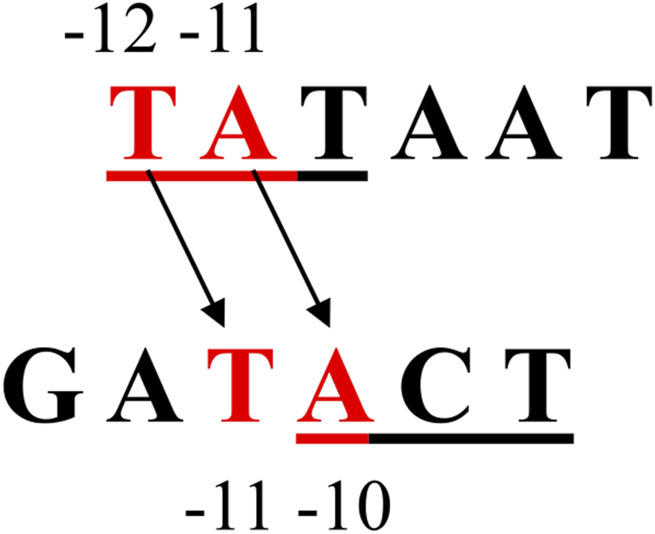
The location of the bases of the −10 region in the consensus promoter and the corresponding base arrangement in this region of the T7 phage A1 promoter. The apparent shift of the −10 sequence from −12 to −7 in the consensus promoter to −13 to −8 in the A1 promoter of T7 phage causes the highly conserved TA dinucleotide to be offset from the TA position in the consensus promoter (−12, −11) and occupies the position −11, −10.

Similar experiments were performed with the A1_−10_ and A1_1/2−10_ promoter fragments changed within the −10 hexamer. In this case, complete or partial changes in the sequence greatly weaken the binding of such DNA to RNA polymerase (Figure 4A). All the changes introduced were limited to hexamers, so they did not affect the sequences separating the −35 and −10 regions, nor did they affect the distance of these hexamers from the transcription start site (+1). Both hexamers remained at the typical distance for the unchanged A1 promoter (17 bp). Analysis of binary complexes with fragments A1_−10_ and A1_1/2−10_ indicates that the change in the “lower” part of the hexamer sequence −10 (bases −10, −9, and −8) reduces the efficiency of complex formation by about 70%. The additional base change −13, −12, −11 (the completely changed hexamer −10) reduces the complexing efficiency only by a further 13%. This suggests that the modification has a greater effect the “lower” part of the region than the “upper” part and reflects the differential function of both parts of this hexamer in the interaction with the enzyme. This suggestion seems to contradict the information that the −12, −11, and −10 bases of the −10 hexamer are crucial (Malhotra et al., 1996; Roberts and Roberts, 1996; Dombroski, 1997; Strainic et al., 1998; Fenton et al., 2000; Fenton and Gralla, 2001; Guo and Tullius, 2003) and are involved in interactions with aromatic amino acids located in domain 2.4 of the sigma subunit. The lower part of this consensus hexamer plays a less important role in the formation of specific interactions as double-stranded DNA but participates in the stabilization of the open complex. The process of DNA “opening” begins in the “upper” part of the −10 hexamer and extends towards the “lower” sequence (towards the transcription start site) and includes subsequent bases of this hexamer (Malhotra et al., 1996; Fenton et al., 2000). So, one would expect a change within the −10 to have a dramatic effect both due to the attenuation of interactions with the double-stranded determinant of the hexamer and the impairment of the single-stranded DNA contribution, which has indeed been demonstrated. We suggested a reasonable proposal to explain such a strong influence of base −8, −9, −10 modifications on the binding of RNA polymerase: there may be a change from the adenine to guanine at the −10 position. If the A1 promoter functioned like the TATAAT consensus promoter, then the “lower” base triplets of the −10 hexamer of the A1 promoter should contribute much less to polymerase binding than the “top” base triplets. In the case of the A1 promoter, however, changing the first 3 bases of the hexamer from the side of the transcription start site causes binding attenuation by about 70%, while the remaining three attenuate it by only about 10%–15% (Figure 3B). So, a promoter sequence element that could be responsible for this effect, this should be the base located downstream of the −10 hexamer of the A1 promoter, in particular the adenine at the −10 position. Shifting the consensus hexamer from position −12 to −7 to position −13 to −8 for the A1 promoter generates a new location of the highly conserved TA pair at position −11, −10 of the A1 promoter (Figure 10).

Being key to the function of the promoter sequence, the consensus promoter adenine −11 currently occupies position −10 in the A1 promoter of phage T7. So, it must perform the same functions as the consensus promoter adenine −11. Relevant domain RNA polymerases recognizing the TA pairs (−12, −11) of the consensus promoter, for stereochemical reasons, must recognize, in the case of the A1 promoter, the TA pairs at −11 and −10. Thus, it is understood that changing the adenine −10 of the A1 promoter also induces drastic changes in promoter function, such as modifying the adenine −11 of the consensus promoter. Changing the −10 base from A to G affects, in the case of the partially altered hexamer −10 (A1_1/2−10_), not only the recognition of the promoter sequence and the “opening” of the DNA, but also the interaction of the bases with the amino acids of the σ subunit and the stability of the open complex. The consequence could therefore be the lack of close contact of the modified hexamer −10 with the RNA polymerase. In such a case, the *Bgl*II sequence present in this region of the modified promoters would be accessible to the restriction enzyme. Such a result is shown in Figure 4, which shows that the action of the *Bgl*II enzyme on the complex binary generates a fragment of about 70 bp. Based on Figure 4, it is impossible to determine whether the 2 fragments produced by the *Bgl*II enzyme remain bound to the RNA polymerase, because the lengths of these fragments are similar. At the same time, it can be concluded that also in the case of a partially altered −10 region, DNA cleavage in the complex takes place. This result would be consistent with experiments showing that mutations within the −10 (−11/–12) region of the *S. typhimurium trp* promoter caused a significant reduction in the protection of this region by RNA polymerase, making it accessible to the restriction enzyme (Oppenheim and Yanofsky, 1989). The fact that the restriction enzyme has access to the *Bgl*II sequence (AGATCT; positions −9 to −4) does not exclude the possibility that a binary complex, even an intermediate or open complex, is formed with low efficiency. It only means that in such a case an equilibrium is established in which the type of complex in which the −10 sequence is not bound to the enzyme has the advantage.

Transformation from the binary to ternary complexes during transcription verifies the assumption that promoter fragments modified in hexamer (−35 and −10) are bound in active open complexes. The ternary complex contains, besides DNA and the enzyme, the nascent RNA molecule. At this stage the enzyme RNA polymerase also lacks a sigma subunit. In our work, transcription was carried out for all modified DNA promoters and the control A1 promoter of T7 phage in a controlled manner, so the reaction product was 11-base RNA (Figures 8A, B). The results of the experiment presented in Figure 8 clearly illustrate the differences in transcription between the modified fragments A1_−35_, A1_−10_, A1_1/2−10_ and the wild-type A1 promoter, and perfectly complement also the conclusions obtained from other experiments. Namely, it is striking that the A1_−35_ fragment (containing the altered −35 region) forms binary complexes very efficiently but has a very low transcription efficiency. In this case, the altered −35 sequence does not seem to interfere with the formation of the open complex, but it fails in the early phases of productive transcription. The modification of the −35 sequence makes the transcription attempt on this promoter lead to a significant reduction in the total radioactivity, corresponding to the binary and ternary complexes. In the case of the unmodified A1 promoter of T7 phage, all radioactive DNA from the binary complex was found in a triple complex position. However, in the case of a promoter containing an altered sequence of the −35 region, we observe a complete disappearance of radioactivity at the position of the binary complex. This radioactivity, however, appears only in a negligible amount in the position of the ternary complex. This clearly shows that a promoter of this type has a significantly reduced ability to initiate RNA synthesis, and the binary complex after the addition of Mg^2+^ ions, nucleotides, and ApUpC becomes sensitive to heparin, and it breaks down. The conducted experiments therefore led to the conclusion that the change in the hexamer −35 sequence only slightly impairs the functioning of the promoter at one of the early stages of initiation, i.e., the formation of at first closed and then open complexes. It dramatically reduces, however, the probability of further steps in transcription initiation, by acting on some of the further discrete steps in the process. The fact that the complex formed with the fragment lacking the −35 consensus sequence is very stable to heparin suggests that it is an open complex. Also, experiments with chemical reagents that modify the bases in single-stranded DNA, e.g., potassium permanganate (Łoziński and Wierzchowski, 2005), clearly show that the mentioned complex is open (Figure 8). This is also supported by the result of digestion of the complex with RNA polymerase with the enzyme restriction *Eco*RI (Figure 4). The fact that this sequence within hexamer −35 is accessible to the restriction enzyme, and that both 96 and 29 bp cleavage products remain associated in the complex, indicates that the DNA is in close contact with the protein in regions outside the −35 hexamer. The shorter DNA fragment (29 bp) remains strongly associated with the sequence located upstream of the −35 region of the promoter, while the “lower” fragment (96 bp) is associated with the sequence located downstream of the −35 hexamer. If the complex in question was a closed complex, then it would immediately disintegrate under the influence of heparin. Moreover, the weak protection downstream of the −35 region suggests that a longer DNA fragment would be released. This part of the closed-complex promoter shows only two limited DNA protection sites. The longer DNA fragment produced by the *Eco*RI enzyme can only remain bound in the complex if the enzyme completely encloses the DNA, providing full protection typical of the complex open (Schickor et al., 1990). Also in favor of this complex being more advanced in the process of transcription initiation than the closed one are the kinetic and structural analyses of the intermediate transcriptions (McClure and Cech, 1978; Schickor et al., 1990), which showed that only heparin-stable open complexes can exist at 37°C. In this case, both the closed and the intermediate complexes are rapidly isomerizing into the open complex intermediates. Also, the transcription attempt, after adding Mg^2+^ ions and nucleotides, suggests that it must be an open complex, subtracting the transcription attempt, because only in this case is the destabilization of the binary complex and the lack of transformation into a ternary complex justified.

Experiments performed on the A1 promoter of phage T7 modified in the hexamer −35 suggest that the most important function of this hexamer is therefore carrying the complex through the early phase of the synthesis of the first phosphodiester bonds of the forming transcript and transformation into an elongation complex. In our case, the open complex is formed by the interaction of the −10 hexamer and non-specific interactions in the −35 region, especially interactions of the sequence located upstream of this hexamer. These experiments confirm that not just the rank of the hexamers is different in RNA polymerase binding and transcription, but also both of these promoter-important sequences perform distinct and complementary functions. Hexamer −10 is absolute necessary for both RNA polymerase binding and all subsequent transcription steps. This is related to the highly specific protein-DNA interactions necessary for the stabilization of the open complex and, above all, to the function of opening the DNA at the stage of creating the open complex (Werel et al., 1991). Warman et al. (2021) also confirmed the unique role of this promoter element. They emphasized that other promoter sequences stabilize polymerase RNA binding, and the −10 motif also facilitates DNA opening and transcription initiation; other promoter sequences are ineffective, without a rightly positioned −10 element. Finally, they showed that the −10 element with appropriate symmetry can function independently to drive divergent transcription (Warman et al., 2021). On the other hand, Einav and Phillips (2019) in their work highlighted the multivalent model of gene expression, where the avidity between −35/–10 elements as well as the independence of the UP/spacer/background interactions were incorporated. An avidity means that when RNAP is singly bound to either the −35 or the −10 sites, it is much more likely (compared with unbound RNAP) to bind to the other site (Einav and Phillips, 2019).

Our work suggests that the role of the −35 region is of a “quantitative” nature, which is necessary to ensure adequate stability complex at the stage of transformation of a binary complex into a ternary complex. Replacing the–35 hexamer with a completely different sequence ensures, due to non-specific interactions, the formation of a sufficiently stable binary complex, but it does not ensure permanent contact of this sequence with the appropriate domain of RNA polymerase during the movement of domains relative to each other, during the formation of transient, unstable complexes associated with the early stages of RNA synthesis. Weak interactions of the modified hexamer −35 with the appropriate domain of the RNA polymerase break this contact during the transcription attempt and expose the β′ subunit to heparin, which leads to the dissociation of the complex. This is confirmation, alongside the analysis of abortive transcription products (Metzger et al., 1993), a single-molecule FRET experiment (Kapanidis et al., 2006), and magnetic tweezer transcription experiments (Lerner et al., 2016), of Straney and Crothers’ hypothesis about the occurrence of the stressed intermediate stage in the early phase of transcription initiation (Straney and Crothers, 1987). This stage is the result of the displacement of RNA polymerase domains in the phase preceding the formation of the elongation complex. It was shown that loss of specific protein-DNA interactions results in an abrupt transition to nonspecific electrostatic interactions (von Hippel, 2007; Rhodius and Mutalik, 2009). Saecker et al. (2021) using cryo-electron microscopy and single point mutations, found that small alterations in the sequence or size of the transcription bubble trigger global changes in RNAP-DNA interactions and in DNA base stacking (Saecker et al., 2021).

The results of the conducted experiments confirming the additive effect of the sequence located upstream of the −35 region and the −35 hexamer in the first phase of transcription initiation, i.e., at the stage of promoter localization and formation of subsequent initiation complexes. The DNA fragment containing the completely altered hexamer −35 is able to form complexes with RNA polymerase up to and including the open complex stage. The natural consequence of this is that promoter recognition is sequential in nature, with the sequence located upstream of the −35 hexamer predominating rather than the −35 region itself. This suggestion has already been confirmed by analyses of the binding kinetics of RNA polymerase with the T7 phage A1 promoter using synchrotron radiation (time-resolved X-ray-generated hydroxyl radical footprinting) (Sclavi et al., 2005).

## Data Availability

The raw data supporting the conclusion of this article will be made available by the authors, without undue reservation.
